# A 20-year multicentre outcome analysis of salvage mechanical circulatory support for refractory cardiogenic shock after cardiac surgery

**DOI:** 10.1186/s13019-016-0545-5

**Published:** 2016-11-08

**Authors:** Maziar Khorsandi, Scott Dougherty, Andrew Sinclair, Keith Buchan, Fiona MacLennan, Omar Bouamra, Philip Curry, Vipin Zamvar, Geoffrey Berg, Nawwar Al-Attar

**Affiliations:** 1 Department of Cardiothoracic Surgery, Royal Infirmary of Edinburgh, 51 Little France Crescent, old Dalkeith road, Edinburgh, EH16 4SA UK; 2 Department of Cardiology, Ninewells University Hospital, Dundee, DD2 1UB UK; 3 Department of Cardiothoracic Surgery, Golden Jubilee National Hospital, Agamemnon street, Clydebank, Glasgow, G81 4DY UK; 4 Department of Cardiothoracic Surgery, Aberdeen Royal Infirmary, Burnside house, Cornhill road, Aberdeen, AB25 2ZR UK; 5 Medical Statistics, Trauma Audit & Research Network, Salford Royal NHS foundation trust and the University of Manchester, Manchester, M6 8HD UK

**Keywords:** Extracorporeal circulation, Heart-assist devices, Post-cardiotomy, Shock

## Abstract

**Background:**

Refractory post-cardiotomy cardiogenic shock (PCCS) is a relatively rare phenomenon that can lead to rapid multi-organ dysfunction syndrome and is almost invariably fatal without advanced mechanical circulatory support (AMCS), namely extra-corporeal membrane oxygenation (ECMO) or ventricular assist devices (VAD). In this multicentre observational study we retrospectively analyzed the outcomes of salvage venoarterial ECMO (VA ECMO) and VAD for refractory PCCS in the 3 adult cardiothoracic surgery centres in Scotland over a 20-year period.

**Methods:**

The data was obtained through the Edinburgh, Glasgow and Aberdeen cardiac surgery databases. Our inclusion criteria included any adult patient from April 1995 to April 2015 who had received salvage VA ECMO or VAD for PCCS refractory to intra-aortic balloon pump (IABP) and maximal inotropic support following adult cardiac surgery.

**Results:**

A total of 27 patients met the inclusion criteria. Age range was 34–83 years (median 51 years). There was a large male predominance (*n* = 23, 85 %). Overall 23 patients (85 %) received VA ECMO of which 14 (61 %) had central ECMO and 9 (39 %) had peripheral ECMO. Four patients (15 %) were treated with short-term VAD (BiVAD = 1, RVAD = 1 and LVAD = 2). The most common procedure-related complication was major haemorrhage (*n* = 10). Renal failure requiring renal replacement therapy (*n* = 7), fatal stroke (*n* = 5), septic shock (*n* = 2), and a pseudo-aneurysm at the femoral artery cannulation site (*n* = 1) were also observed. Overall survival to hospital discharge was 40.7 %. All survivors were NYHA class I-II at 12 months’ follow-up.

**Conclusion:**

AMCS for refractory PCCS carries a survival benefit and achieves acceptable functional recovery despite a significant complication rate.

## Background

Cardiogenic shock following cardiac surgery can affect as many as 2–6 % of patients undergoing routine surgical coronary revascularization or valve surgery [1–4]. Although the majority of these patients respond to inotropic support and/or intra-aortic balloon pump counter pulsation (IABP) support, 0.5–1.5 % of patients demonstrate a rapid and progressive decline in their haemodynamic parameters in the immediate aftermath of cardiopulmonary bypass [5]. The occurrence of post-cardiotomy cardiogenic shock (PCCS) can be unpredictable and can occur in patients with normal preoperative myocardial function as well as those with pre-existing impaired function [6]. Refractory PCCS leads to vital organ hypoperfusion and is almost universally fatal [4, 7–9] without the use of advanced mechanical circulatory support (AMCS) devices such as extracorporeal membrane oxygenation (ECMO) or ventricular assist devices (VAD).

In our previous study we looked at the outcomes of AMCS utilization at the Edinburgh heart center’s cardiothoracic surgery department (a non-transplant, intermediate-sized, adult cardiothoracic surgery centre) in Scotland [10]. This current multicentre observational study aims to consolidate our previous findings and looks at the 20-year outcomes of AMCS utilization to salvage refractory PCCS patients in all the 3 cardiothoracic surgery centres in Scotland.

## Methods

Scottish adult cardiothoracic surgical services are provided by three regional centres covering a population of 5.2 million individuals [11]. The relevant data was collected from the databases of the Royal Infirmary of Edinburgh (surgical case load ≈ 900/year), the Golden Jubilee National Hospital in Glasgow (surgical case load ≈ 1300/year), and the Aberdeen Royal Infirmary (surgical case load ≈ 500/year). Our inclusion criteria included any adult patient from April 1995 to April 2015 who had received salvage VA ECMO or VAD for PCCS refractory to IABP and inotropic support following adult cardiac surgery. We acquired information regarding the patients’ 12 month follow-up status by accessing the cardiology follow-up clinic letters on the TrakCare^R^ system in Edinburgh, the AMCS database in Glasgow, and through making direct enquiries with the surgeons involved in the long-term outcomes of the patients in Aberdeen via email and telephone communications.

The AMCS devices utilised at the Royal Infirmary of Edinburgh over the defined study period were Levitronix^R^ CentriMag II for ECMO and Medtronic Bio-Medicus^R^ 560 for short-term VAD support. Over the same time period, the AMCS devices used at the Golden Jubilee National Hospital in Glasgow and the Aberdeen Royal Infirmary cardiac surgical units was the CentriMag device for both VA ECMO and short-term VAD support.

## Results

A total of 28 patients met the inclusion criteria with one patient excluded due to lack of recorded information in the TrakCare^R^ database regarding the type of AMCS support used, any potential complications and the short and the long-term outcomes of this individual. Overall, 16 patients from the Royal Infirmary of Edinburgh met the inclusion criteria, 8 patients from the Golden Jubilee National Hospital in Glasgow and 3 patients from Aberdeen Royal Infirmary cardiothoracic surgery unit. The reason why more cases belonged to Edinburgh rather than Glasgow, despite the latter being a larger unit, was because AMCS was rarely used to salvage refractory PCCS patients in the west of Scotland prior to 2007 (the year of the merger between Glasgow Royal Infirmary and the Glasgow Western Infirmary forming the Golden Jubilee National Hospital).

**Fig. 1:**
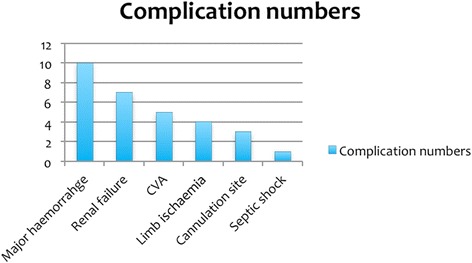
Bar chart illustrating the number and nature of complications within cohort

Of the total 27 patients from the 3 centres, the age range was 34–83 years (median 59 years). There was a large male predominance of 23 (85 %). Four patients (15 %) had undergone re-operative cardiac surgery. One patient (3.7 %) had undergone AMCS following the repair of a traumatic ascending aortic transection after a road traffic accident. Overall, 23 patients (85 %) had received a single run of VA ECMO of which 14 (61 %) had received central ECMO and 9 (39 %) had received peripheral ECMO. Four patients (15 %) had short-term VADs (1 BiVAD, 1 RVAD and 2 LVAD). The mean duration of AMCS was approximately 5.43 days (Range < 1 day–33 days). The most common procedure-related complication was major haemorrhage (37 %). Renal failure requiring renal replacement therapy (26 %), stroke (19 %) and peripheral limb ischaemia (15 %, Fig. 1) were also recorded. Logistic EuroSCORE ranged from 2.08 to 73.26. More detailed patient baseline characteristics are tabulated in Table 1.

**Table 1:** Patient baseline characteristics

	Age & Gender	Date of surgery	Original operation	Duration and Mode of AMCS	AMCS Complication/s	Outcome
Patient 1	76 year old male	2012	Re-do sternotomy and AVR	Salvage peripheral VA ECMO due to postoperative pulmonary haemorrhage and cardiogenic shock	Femoral artery cannulation site pseudoaneurysm	Alive
						NYHA I (No breathlessness of exertion, back to work)
					Major haemorrhage from cannulation site	
Patient 2	40 year old male	2014	Re-do, Re-do sternotomy for type A aortic dissection: Bentall procedure	Salvage RVAD due to VF arrest and severe LVSD after weaning from CPB	Major haemorrhage and re-exploration in the operating theatre	Alive
						NYHA II (Breathless on exertion)
Patient 3	82 year old male	2006	MV Repair and CABG	3 Days	Could not be weaned from ECMO with severe biVent failure and	Died in CTICU
				VA ECMO as unable to wean from CPB		COD: BiVent failure
Patient 4	72 year old Female	2011	AVR	9 Days	Septic shock	Died in CTICU
				VA ECMO as unable to come off CPB	Limb ischaemia	COD: Septic shock
Patient 5	71 year old male	2011	CABG and AVR	2 Days	ECMO cannulation site bleeding and haematoma explored	Died in CTICU
				Peripheral VA ECMO as unable to come off CPB		COD: Shock (unknown cause)
					Renal failure ^a^	
Patient 6	83 year old female	2012	MVR and CABG	<1 Day	None	Died in CTICU
				Peripheral VA ECMO as unable to wean from CPB		COD: BiVent failure
Patient 7	70 year old male	2013	Re-do sternotomy and AVR	33 Days	Major CVA	Died in HDU
				VA ECMO for cardiac failure. Successfully weaned from ECMO		COD: severe Respiratory failure
Patient 8	72 year old male	2013	Re-do sternotomy and AVR	<1 Day	ECMO cannulation femoral artery dissection	Died in CTICU
				VA ECMO after iatrogenic aortic dissection leading to cardiogenic shock during Femoral cannulation for CPB		COD: Major CVA
					Major haemorrhage	
					Major CVA	
Patient 9	51 year old male	2013	Re-suspension of Aortic valve and repair of type A aortic dissection	1 Day	Major cannulation site haemorrhage	Died in CTICU
				Peripheral VA ECMO for cardiogenic shock		COD: Haemorrahgic shock and BiVent failure
Patient 10	34 year old female	2014	IVC Leiomyosarcoma resection	3 Days	None	Died in CTICU
				VA ECMO for postoperative cardiogenic shock for intraoperative MI		COD: BiVent failure from acute MI
Patient 11	65 year old male	2013	CABG	2 Days	Renal failure^a^	Died in CTICU
				Salvage VA ECMO for cardiogenic shock	Hepatic failure	COD: MODS
					Pulmonary oedema	
Patient 12	71 year old male	2015	CABG	3 Days	Major haemorrhag e: Re-opening for bleeding x4	Died in CTICU
				VA ECMO as unable to wean from CPB		COD: biventricular failure and septic shock
					limb ischaemia	
Patient 13	49 year old male	1997	CABG	VA ECMO as unable to wean from CPB	Note recorded	Alive
						(Died 2004)
						NYHA II
Patient 14	69 year old male	2004	MVR and CABG for mitral valve IE	VA ECMO as unable to wean from CPB	CVA and seizures	Alive
					Renal failure ^a^	NYHA II
Patient 15	41 year old female	2005	Aortic transection and diaphragm rupture	VA ECMO	Not recorded	Alive
						NYHA I
Patient 16	59 year old male	2006	Type A aortic dissection	2 Days	Not recorded	Died
				Peripheral VA ECMO as unable to wean from CPB		COD: Bivent failure
Patient 17	21 year old male	2014	AVR	3 days	ECMO cannulation site bleeding-required re-exploration	Alive
				Peripheral VA ECMO		NYHA I
					Cardiac tamponade	
Patient 18	51 year old male	2014	AVR	6 days	CVA and Seizures	Died in ICU
				Peripheral VA ECMO	limb ischaemia	COD: status epilepticus
Patient 19	46 year old male	2014	CABG	2 days	Major haemorrahage	Died in ICU
				Peripheral VA ECMO converted to central VA ECMO due to peripheral ischaemia		COD: MODS
					Limb ischaemia/compartment syndrome-bilateral fasciotomies	
					Renal failure^a^	
Patient 20	54 year old male	2015	CABG and AVR	3 days	SVT/VT	Alive
				VA ECMO for cardiogenic shock	Major intra-abdominal haemorrhage requiring laparotomy	NYHA II (Neuropathic leg pain)
					Limb ischaemia	
Patient 21	56 year old male	2015	AVR	3 days	CVA (occipital infarcts)	Alive
				Peripheral VA ECMO for cardiogenic shock		NYHA I (Visual difficulties)
Patient 22	64 year old male	2015	AVR	1 day	Vasoplegia	Died
				VA ECMO	MODS	COD: AV dissociation
Patient 23	52 year old male	2015	CABG	1 day	MODS	Died
				VA ECMO		COD: MODS
Patient 24	64 year old male	2015	AVR	7 days	None	Alive
				VA ECMO		NYHA I
Patient 25	50 year old male	2014	AVR	23 days	Renal failure^a^	Alive
				BiVAD		NYHA I
					Haemothorax/mediastinal collection requiring re-operation	
Patient 26	54 year old male	2015	Bentall’s procedure and CABG surgery	2 days	Hepatic failure	COD: MODS
				LVAD acute LV failure	Renal failure please^a^	
Patient 27	61 year old male	2003	CABG	11 days	Respiratory failure	Alive
				LVAD for acute LV failure	Renal failure^a^	NYHA II

*Abbreviations*: *ACS* Acute coronary syndrome, *AF* atrial fibrillation, *AMCS* Advanced mechanical circulatory support, *AVR* Aortic valve replacement, *CABG* Coronary artery bypass grafting surgery, *CPB* Cardiopulmonary bypass, *COD* cause of death, *BiVent failure* BiVentricular failure, *MVR* Mitral valve replacement, *IE* Infective endocarditis, *CVA* Cerebrovascular accident, *IVC* Inferior vena-cava, *NYHA* New York Heart Association, *CTICU* cardiothoracic Intensive care unit, *HDU* High dependency unit, Implantable cardioverter defibrillator, *MI* Myocardial infarction, *LVSD* Left ventricular systolic dysfunction, *TVD* triple vessel coronary artery disease, *LV* left ventricular, *MR* Mitral regurgitation, *PVD* Peripheral vascular disease, *MODS* Multi-organ dysfunction syndrome, *VF* Ventricular fibrillation, *VAD* Ventricular assist device, *VA* Veno-Arterial

^a^All patients with renal failure required renal replacement therapy

**Fig. 2:**
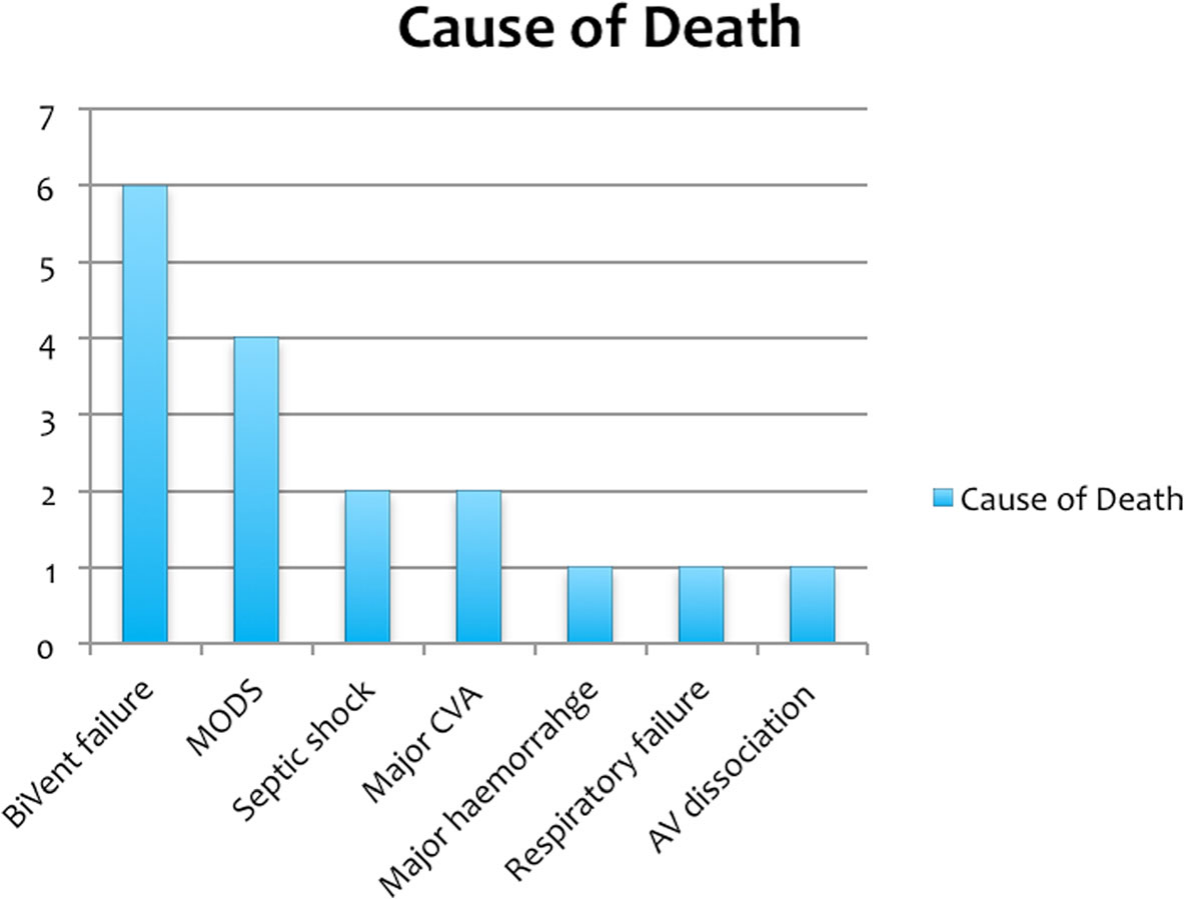
Bar chart illustrating the number and causes of death within cohort. AV: atrioventricular

**Fig. 3:**
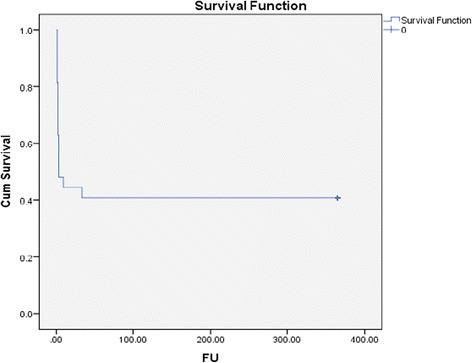
Kaplan-Meier curve of survival, *x*-*axis* represents follow-up (F﻿U) in days and *y*-*axis* represents cumulative survival (Cum survival)

The most common cause of death (COD) was refractory biventricular failure that failed to recover sufficiently to allow weaning from AMCS (22.2 %, Fig. 2). In these patients care was withdrawn. One patient died due to a combination of biventricular failure and haemorrhagic shock and another patient died from a combination of biventricular failure and septic shock whilst on VA ECMO. The survival rate to hospital discharge was 40.7 % (Fig. 3). The follow-up data showed that the survivors were all NYHA class I-II functional status at 12 months.

### Statistical analysis

Statistical analysis was performed using the Fisher’s exact and Pearson’s chi^2^ tests. Univariate analysis was performed. Table 2 demonstrates the baseline statistics data and the analytical methods used in this study.

**Table 2:** Demonstrates variables used for statistical analysis. Fisher’s exact test and Pearson’s chi test (Log. EuroSCORE) were utilized for statistical analysis

Factors attributed to mortality and statistical analysis				
Characteristics analyzed	Alive	Dead	Odds ratio (95 % Conf. interval)	*p*-value
Age (years)				
0–65	8	10	2.8 (0.362853–33.74714)	0.24
> 65	2	7		
Gender				
Male	9	14	1.928571 (0.1270413–112.3145)	0.5
Female	1	3		
Type of center				
Transplant	4	5	0.625 (0.0921389–4.488993)	0.44
Non-transplant	6	12		
Prev. cardiac surgery				
Re-do surgery	2	2	0.5333333 (0.0335265–8.873345)	0.48
First time surgery	8	15		
Surgical complexity				
Isolated surgery	6	10	1.05 (0.1662785–7.107629)	0.64
Complex surgery	4	7		
Type of Support				
VAD	3	1	0.1458333 (0.0026189–2.352801	0.13
ECMO	7	16		
Duration of Support				
0–7 days	8	15	0.5333333 (0.0335265 8.873345)	0.47
> 7 days	2	2		
Support complications				
Major haemorrhage	5	5	0.4166667 (0.0620347–2.804408)	0.25
No major haemorrhage	5	12		
Major CVA	1	4	2.769231 (0.2140667–151.2664)	0.37
No major CVA	9	13		
Renal failure	3	4	0.7179487 (0.0910803–6.420841)	0.52
No renal failure	7	13		
Log. EuroSCORE				
0–10	1	3		0.36 (Pearson’s chi^2^ test)
10–20	1	6		
> 20	4	3		
Score not available	4	5		

Table information: Prev.cardiac surgery denotes whether the patient had had previous cardiac surgery through median sternotomy (i.e. redo surgery). Isolated surgery refers to whether the operation was isolated coronary artery bypass grafting surgery (CABG) or single valve surgery. Complex cardiac surgery refers to combined valve, CABG and/or aortic surgery. Type of center denotes whether the operating hospital in which the operation was performed was a cardiopulmonary transplant center. Log. EuroSCORE refers to logistic EuroSCORE

## Discussion

Our study demonstrates that AMCS used for the treatment of refractory PCCS can lead to good outcomes for a significant number of patients, with 40.7 % surviving to hospital discharge and all surviving patients were graded as either NYHA class I or II at 12 months’ post-discharge. Without AMCS, it is likely that the vast majority of these patients would have died. Ours is also the first multi-centre study of its kind to emerge from the UK and one of the few studies to examine functional outcomes post AMCS utilisation for refractory PCCS.

Recent evidence has demonstrated that modern, continuous-flow AMCS devices, such as the CentriMag^R^ that was used in our centres, can lead to improved survival in patients with PCCS [12–14]. In the largest cohort, Hernandez et al. [3] collated data from 5735 patients who underwent salvage VAD for refractory PCCS. They reported a 54.1 % survival rate to hospital discharge and concluded that VAD is a valuable, life-saving therapeutic manoeuvre. By comparison, the survival rate in our study was lower but firm conclusions are difficult given the low number of patients in our cohort. However, other smaller studies (relative to the Hernandez study) [5, 15–18] all using either ECMO or VAD for refractory PCCS, reported less impressive survival to hospital discharge rates of 24.8 %–37 % and a 5 year survival of 13.7 %–16.9 %. Unfortunately, we do not have long-term survival data as many of the survivors were ultimately discharged from the outpatient clinics when no further medical or surgical interventions were required, hence longer term follow up data post out-patient clinic discharge had not been recorded in the database.

We identified advanced age to be a factor leading to an adverse outcome, although again, owing to our smaller numbers, this did not reach statistical significance. Most (64 %) of the survivors were under 60 years of age. Furthermore, the emergent nature of surgery and pre-existing, preoperative severe left ventricular impairment were also identified as probable factors leading to an adverse outcome.

Evidence suggests that early device implantation [6] and appropriate patient selection through a multidisciplinary team approach is paramount to an optimal outcome [10]. There are no national or local protocols for identifying suitable patients for AMCS with refractory PCCS in Scotland: instead, decisions are based on a case-by-case assessment involving a multidisciplinary team (cardiac surgeon, department head, anaesthetist, and perfusionist) in each of the three hospital sites. We continue to believe that this is the best approach to patient selection rather than a standardised algorithmic approach because it ensures an ethically appropriate decision for the patient whilst optimising the cost-benefit equation. The decision regarding when to initiate AMCS support was made for most patients whilst in theatre in those whom weaning from CPB was not possible, although a few were commenced AMCS whilst in ICU. The time to AMCS and how this correlates to survival is an important variable that regrettably was not consistently recorded in our patient cohort.

AMCS devices are expensive [9, 19, 20] and this, coupled with a potentially prolonged length of stay in ICU, means that cost is an important factor in the decision-making process, particularly within the UK NHS. Indeed, decision-makers have opted to centralise AMCS funding to a restricted number of the larger cardiothoracic centres [21], invariably depriving other units of this potentially life-saving resource. Understandably, this has led to expressions of consternation [21]. In our cohort, the longest duration on AMCS was 33 days (patient 7). This patient was successfully weaned from VA ECMO but died whilst in critical care from a stroke, which may have been a complication from AMCS employment.

The NYHA functional outcomes for our patients were also very positive. Unfortunately, many previous AMCS studies for refractory PCCS do not report such findings, although we did identify two studies, each with similar outcomes to ours. Ko et al. [17] detailed a cohort of 76 patients undergoing ECMO support for refractory PCCS. They reported that all survivors were of NYHA classes I or II at 32 +/− 22 month follow-up. Pennington et al. [15] reported on refractory PCCS support with VAD and found that all survivors were “leading active lives”. In 72.7 % of their survivors, ejection fraction had normalized on follow-up echocardiography.

Clearly, given that we only identified 27 patients undergoing AMCS over a 20-year period, and despite our pooled hospital case volume, we acknowledge that the Scottish approach to institution of AMCS for refractory PCCS has been relatively conservative. This can partly be explained by the fact that salvage AMCS was not employed in the west of Scotland until 2007. Also, our general approach to institution of AMCS dictates that such modalities are instituted only if there is a reversible cause of the cardiogenic shock, which is reflected by our reasonable survival rate. Other possible reasons for underutilization may include: scarcity of resources, prohibitive costs, and lack of consistent evidence for the benefit of AMCS.

The decision to institute AMCS must also be balanced with due consideration of the associated risks of this invasive modality, many of which are potentially life-threatening. Common device-related complications include: haemorrhage, thrombus formation and embolization, stroke, device-related infection, limb ischaemia, and multi-organ dysfunction syndrome/failure [1, 2, 15, 17, 22, 23]. In our cohort, the most common procedure-related complication was major haemorrhage. Renal failure requiring renal replacement therapy, stroke, and peripheral limb ischaemia also occurred with comparable rates to previous studies.

Given the scarcity of donor hearts in the UK, research continues to focus on implantable AMCS devices as a bridge to recovery, bridge to transplant, or as destination therapy [19]. However, none of our patients were transplanted during the study period and none had implantable long-term VADs.

Finally, this study is limited by the small number of subjects (as previously discussed) and its retrospective nature. It nevertheless reaffirms the findings of our previous study, which reported a good survival rate and acceptable quality of life for patients who received AMCS for refractory PCCS and survived to hospital discharge.

## Conclusions

AMCS devices can be used to salvage a significant proportion of patients with refractory PCCS who would otherwise not survive. These patients are also likely to enjoy a reasonable quality of life. However, ACMS devices are associated with high rates of severe, systemic and device-related complications as well as being costly. Multidisciplinary teams experienced with patient selection and decision-making are imperative to help ensure appropriate use of AMCS and the best patient outcomes.

## Abbreviations

ACS: Acute coronary syndrome
AF: atrial fibrillation
AMCS: Advanced mechanical circulatory support
AVR: Aortic valve replacement
CABG: Coronary artery bypass grafting surgery
CPB: Cardiopulmonary bypass
COD: Cause of death
BiVent failure: BiVentricular failure
MVR: Mitral valve replacement
IE: Infective endocarditis
CVA: Cerebrovascular accident
IVC: Inferior vena-cava
NYHA: New York Heart Association
CTICU: Cardiothoracic Intensive care unit
HDU: High dependency unit
ICD: Implantable cardioverter defibrillator
MI: Myocardial infarction
LVSD: Left ventricular systolic dysfunction
TVD: triple vessel coronary artery disease
LV: left ventricular
MR: Mitral regurgitation
PVD: Peripheral vascular disease
MODS: Multi-organ dysfunction syndrome
VF: Ventricular fibrillation
VAD: Ventricular assist device
VA: Veno-Arterial
